# A Literature Review on the Foundations and Potentials of Digital Teaching Scenarios for Interprofessional Health Care Education

**DOI:** 10.3390/ijerph17103410

**Published:** 2020-05-14

**Authors:** Johannes Grosser, Martina Bientzle, Joachim Kimmerle

**Affiliations:** 1 Knowledge Construction Lab, Leibniz-Institut fuer Wissensmedien, 72076 Tuebingen, Germany; j.grosser@iwm-tuebingen.de (J.G.); m.bientzle@iwm-tuebingen.de (M.B.); 2 Department for Psychology, Eberhard Karls University, 72076 Tuebingen, Germany

**Keywords:** Medical education, interprofessional collaboration, health care, digital learning platforms, videos

## Abstract

The health care system is increasingly complex and specialized, but it presents the actors involved with the challenge of working together in interprofessional teams. One way to meet this challenge is through interprofessional training approaches, where representatives of different professions learn together with learners of other professions. This article contributes to the question of how interprofessional teaching in health care education can be designed with a low threshold by using digital media. We focus on learning with digital learning platforms and learning with videos. Based on existing empirical findings, these approaches are discussed in terms of their potential and limitations for interprofessional teaching. In particular, we examine how these approaches influence the core competence domains of interprofessional collaborative practice. Digital collaborative learning platforms are suitable for teaching interprofessional competences, since they enable social and professional exchange among learners of different professions. Videos are suitable for imparting medical declarative and procedural knowledge. Based on these considerations, the use of videos in combination with interaction possibilities is presented as a didactic approach that can combine the aspect of knowledge transfer with the possibility of interprofessional computer-based collaboration.

## 1. Introduction

The demand to implement interprofessional training concepts in medical education has been repeatedly raised by various actors [1,2,3]. Successfully meeting this demand requires well-considered and scientifically sound didactic concepts in addition to the establishment of appropriate structures. With the present article we want to contribute to the question of how interprofessional teaching in medical education can be shaped by the appropriate use of digital media. First, we present current challenges in the implementation of interprofessional training concepts. We then describe selected theoretical considerations and empirical findings that are of central importance for digital interprofessional training concepts. In the course of this presentation, we explain and evaluate these didactic approaches with the aim of developing concepts that can prepare future doctors and trainees in health care professions for efficient and smooth interprofessional collaboration in their everyday work.

## 2. Current Challenges

### 2.1. Specialization and Interprofessional Collaboration

The environment in which students of medicine and other health professions work is becoming increasingly challenging [4,5]. Medical and technical progress is constantly expanding the repertoire of diagnostic procedures, interventions, and therapeutic approaches, which has led to the health care system becoming ever more specialized and differentiated. As a result, it has become more efficient and offers patients better medical care. At the same time, these developments have led to an increased complexity of the health care system [6]. In addition to doctors, numerous other specialized professions with different professional backgrounds are active in the health care system [7,8]. In the clinical areas, representatives of a wide range of health care professions are active, who support the medical staff in their treatment. These include nurses, physiotherapists, speech therapists, and many others. Even though interprofessional collaboration in medicine has always been necessary for good patient care, the increasing complexity of the health care system makes the establishment of coordinated collaboration and communication among the various specialists more relevant today than ever. 

Members of different health care professions may hold different views, perspectives, and attitudes [9,10,11]. In general, people tend to form groups based on social categories, such as gender, ethnicity, or, in the present case, professional identity [12]. This has an impact on their self-concepts and sense of belonging to certain groups, which can lead to some kind of “tribalism” and, as a consequence, to failures in communication [13]. All health care professions aim for effective patient care, but tribalism and persisting professional stereotypes [14,15] may impair successful teamwork [16,17]. Everyday clinical practice shows that difficulties often arise in interprofessional communication and collaboration [3].

### 2.2. Interprofessional Education

Medical students are facing growing challenges due to these changes in the health care system. While the professional demands of medical education have remained consistently high, the ability to work in a team and to work together with other professions are becoming increasingly important [3]. A key to successfully meeting these challenges is offered by interprofessional training approaches, such as those already prevalent in the Scandinavian- [18,19] and English-speaking countries [20,21]. In other countries, like Germany, there is also a call for more interprofessional training approaches to be implemented in medical education, and for human and dental medicine courses to be networked with courses in nursing, therapy, and midwifery [22,23]. Students may enter their academic studies with negative stereotypes of other health care professions, which may influence their future work behavior [24] and which is also reinforced by educators [25]. Thus, it is important to implement positive interprofessional experiences and scenarios in the curriculum in order to prevent tribalism among health care students [26]. An early integration of interprofessional learning sessions are therefore helpful to reduce professional stereotypes among health care students [27]. Interprofessional education should be implemented through teaching situations in which learners from at least two health care professions actively learn together. In interprofessional courses, learners should be given the opportunity to acquire and develop not only specialist knowledge, but also social skills and beliefs that are conducive to well-coordinated interprofessional collaboration. To this end, a panel of experts from the Interprofessional Education Collaborative has proposed a framework model, whereby four core competence domains are to be counted among interprofessional collaborative practice [28]:

(1) Values and ethics. Values and ethics are part of the professional identity of both practitioners and learners. Older approaches to professional identity have been criticized for being selfish and thus tending to create barriers between professions, which is seen as an obstacle to improving health care [29]. Therefore, new patient-oriented and society-centered values are proposed, based on the common goal of improving health care overall. The associated professional ethics thus reflect the shared commitment of different professions to creating safer, more efficient and effective health care systems. This is accompanied by a consistent demonstration of mutual trust and respect in the communication among various professionals [30], whose establishment we already consider to be particularly important for training health care professionals.
(2) Awareness of profession-specific roles, competences, and responsibilities. The roles and responsibilities of the individual professions are defined by legal requirements, but this can vary depending on the specific care situation. Therefore, a concrete understanding of the boundaries of expertise between the professions and knowledge of competences of the professions is crucial and should be practiced and refined in continuous interprofessional education as well as in practice [31,32].
(3) Ability for interprofessional communication. A study by Suter et al. identified the ability of interprofessional communication as a core aspect of interprofessional collaboration [33]. This often faces the challenge that different professions often use different terms, thus preventing effective interprofessional care. In order to guarantee this care, the aim of interprofessional education should be to establish a common interprofessional language, which is trained through appropriate training and practice opportunities.
(4) Ability for interprofessional teamwork. Wherever members of different health care professions work together to achieve common goals for the care of patients, they must demonstrate teamwork skills. Accordingly, they must learn to bring their own expertise into a complex system that achieves better results through individual contributions. Focusing on patient care and dealing openly and constructively with conflicts within the team through effective interprofessional communication and joint problem solving strengthens the ability to work together and form a more effective team.

Even though interprofessional training approaches have a great potential for the acquisition of relevant competences of interprofessional collaboration, their implementation in practice is very costly and often fails because the necessary structures have not yet been established across the board. In addition, most health-related training and study courses already have a high time load, which makes it extremely difficult to implement further content in studies and training [34,35]. This results in the challenge of integrating interprofessional teaching concepts into existing curricula across training and institution boundaries.

It must be critically noted that all the demands to strengthen interprofessional teaching are only supported by mixed empirical evidence. For example, the introduction of interprofessional learning methods has led to better treatment results and greater patient satisfaction in several meta studies [36,37]. However, a Cochrane Review of nine studies reports that there is no or at best only a very weak correlation between better interprofessional competence and better clinical results [38]. It is therefore questionable whether a far-reaching investment, such as the creation of cross-organizational training units for the implementation of interactive interprofessional learning scenarios in medical education, will actually lead to better interprofessional communication and collaboration in clinical practice in the long term. One possibility would initially be to integrate interprofessional teaching in medical education with less investment-intensive and low-threshold offerings. To promote interprofessional teaching, digitalization offers a number of possibilities, which are presented in the following section. 

## 3. Didactical Approaches

### 3.1. Digital Interprofessional Teaching: The Application of Learning Platforms

A potentially effective strategy to overcome the challenges mentioned in the previous section and to address the four core competence dimensions described by the Interprofessional Education Collaborative is the implementation of digital teaching concepts. These concepts use digital, online-based resources to structure and deliver learning content and provide learners with networking and interactive exchange opportunities. [39]. A corresponding possibility is offered by digital platforms, as they are known from the context of social media on the Internet [40,41,42,43]. These enable users to access digital media, exchange and share information, and discuss profession-specific approaches, independent of time and location. The use of social media platforms is very common among students [44], and these platforms are often also used for educational purposes [45]. This also applies to medical educational contexts [46], where social platforms are a widely used tool for professionalization [47]. Social media platforms could enable future doctors to develop an awareness of the responsibilities and limitations of their own and other professions, and provide an easy way to apply interprofessional learning into medical education [48]. Nevertheless, these social media platforms are rarely used for such interprofessional applications [44,49].

Therefore, learning management systems were explicitly designed to support such university teaching scenarios. Various forms of online learning have been shown to be effective for the transfer of knowledge and the development of interprofessional competences. They enable learners to gather their own knowledge and exchange opinions and experiences regardless of place, time, educational progress, or profession [50]. Learning platforms that involve users more actively are also increasingly popular [51,52]. 

The analysis of the learning behavior of a large sample of medical students on the digital learning platform Amboss has shown that active engagement with the learning material is also key to knowledge acquisition on digital platforms, such that learners acquired more knowledge when they actively supplemented their learning material with additional information [53]. For interprofessional education, opportunities for the social integration of learners are in the foreground, such as commenting and chat functions, microblogging, forums, and surveys [54,55,56]. An example of low-threshold chat-based learning platforms are electronic communities of practice, in which different medical professions can exchange information via a web-based chat [57]. These self-organized, voluntary associations of different health care providers work in a problem-focused manner and are suitable for different professions to exchange information at eye level. Although such unmoderated exchange platforms often do not show any significant increase in knowledge, they encourage learners to critically reflect on their own level of knowledge [58].

These findings suggest that the design of effective digital learning platforms should be theory-based. Hean, Craddock, and O’Halloran give an overview of the different learning theories from behaviorism to constructivism that can be used to describe online-based processes of interprofessional learning [59]. The approach of Hughes et al. should be emphasized here, who, with reference to the concept of enquiry-based learning, present the ways in which online environments can be conducive to inter-professional medical education [60]. Enquiry-based learning is the term used to describe learning processes that contribute to the discovery of new causal relationships through active, self-directed action by the learner [61]. Central to this is the active participation and exchange among learners and the responsibility of learners for their own learning progress [62,63]. Accordingly, it is assumed that learning takes place as an iterative process in which deeper levels of understanding are gradually reached. As a result, in heterogeneous groups typical of interprofessional training, those with more prior knowledge progress faster in learning and communicate their findings better. This shared knowledge content can then serve as orientation and impetus for the knowledge construction process of learners with less prior knowledge. [60]. Thus, asynchronous online platforms focus on the learners themselves, and allow them to determine the speed of their learning processes themselves.

**Figure 1:**
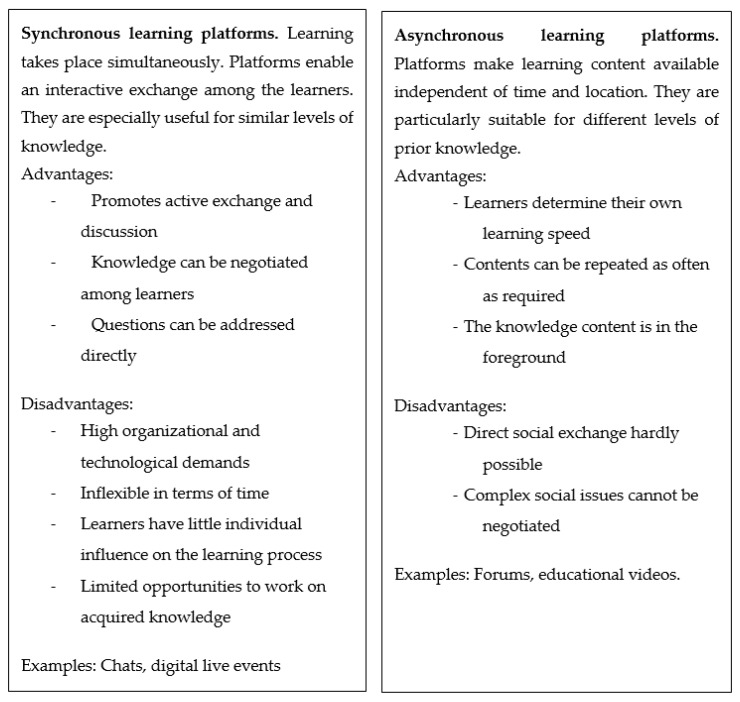
Advantages and disadvantages of synchronous and asynchronous digital teaching aids.

Although asynchronous digital learning platforms in particular are well suited for conveying knowledge content, they quickly reach their limits when it comes to conveying social forms of interaction or values that need to be negotiated in an interactive process among learners, and are therefore less suitable for conveying interprofessional collaboration [64,65]. Accordingly, digital learning formats that are designed for interprofessional purposes should encourage interaction in the learning group [66]. One way to do this is to use synchronous digital formats (see Figure 1 for an overview). An advantage of these teaching formats is that they can also be used informally, and thus stimulate social exchange [45,67]. This can contribute to the development of interprofessional competences and to the development of a positive attitude toward other professions. 

In sum, digital learning platforms are able to address the four core competence domains of interprofessional collaboration. Regarding the development of values and ethics it is important to note that the active interaction on digital learning platforms enables students to share their views and treatment suggestions from their own point of view. Using a digital learning platform allows students to reflect upon their treatment ideas and integrate other people’s ideas in order to provide coordinated health care [68]. Using asynchronous platforms allows learners to spend more time integrating different views, while synchronous platforms allow learners a more direct and vivid discussion [69], which helps to establish mutual trust among various professions [30]. Digital learning platforms can also help learners to raise awareness of profession-specific roles, competencies, and responsibilities by providing reflective questions. Digital learning platforms with students’ active participation can help them to learn about the roles of others and reflect their own role [70]. Learning platforms may strengthen learners’ ability for interprofessional communication. Supportive environments help learners to build trust and encourage active participation and discourse [71]. Synchronous digital learning platforms are particularly suitable for that purpose, as they allow for clarifying misunderstandings immediately. Inconsistencies, in terminology for example, can also directly be addressed by moderators, which would support the establishment of a shared language [72]. Learners’ ability for interprofessional teamwork could be strengthened by the opportunity to cooperate in planning, conceptualization, and coordination of treatments. As a counter model to many hierarchical structures in health care organizations [73], digital learning platforms can be used to establish teamwork of learners on eye-level. This can reduce stereotypes and strengthen the ability to work together and form more effective teams [74]. For synchronous digital formats, there is a danger that the reception of relevant knowledge will be neglected due to lively social exchange. Therefore, they should be complemented with tools and materials that are more suitable for the teaching of specialist knowledge. An appealing and entertaining way of doing this is the use of videos.

### 3.2. Digital Interprofessional Teaching: The Application of Videos

Videos are already widely used in medical education and are very popular with learners, especially because they have established themselves as efficient and clear teaching aids [75]. Numerous studies have shown that watching videos is particularly suitable for acquiring procedural knowledge and, for example, leads to better surgical skills of learners compared to learning in traditional textbook settings [76,77,78,79].

Positive effects on knowledge acquisition from multimedia resources such as videos can be explained by the Cognitive Theory of Multimedia Learning [80]. A basic assumption of the theory is that learners process visual and auditory information separately and that learning can be enhanced by presenting on both channels simultaneously. Since working memory capacity is limited [81,82], the ability to pause and jump forward and backward in videos can help to regulate working memory requirements and thus the learning process [83]. With regard to the acquisition of interprofessional competences, the basic assumptions of Bandura’s social–cognitive learning theory are of particular interest, according to which the learning of new behavior is possible by observing a model and anticipating their behavior [84,85]. Thus, through observation, while observing experts performing a complex task, learners can construct an adequate cognitive representation of that action, mentally imitate that action accordingly, and thereby refine their original mental representation. The observation of successful interprofessional collaboration should therefore have an influence on both the acquisition of procedural knowledge and the attitude toward interprofessional work. These assumptions could be confirmed in a study in which learners from two different professions watched the same video on interprofessional care of a shoulder trauma [86]. This improved the attitudes of physiotherapy students as well as medical students toward interprofessional learning (measured with the learning subscale of the University of the West of England Interprofessional Questionnaire [87,88]) and developed a better knowledge of the competences of the other profession. 

Videos also offer some advantages in terms of conveying knowledge content. They are particularly suitable for the teaching of procedural knowledge, since the time reference allows dynamic actions to be better represented, and thus better understood by learners. Video-based formats therefore clearly stand out from classical text-based teaching [89,90]. This is an advantage in interprofessional teaching settings, where the coordinated action of various protagonists plays a key role. However, as learners often have different levels of prior knowledge, there is a risk that they may quickly be under- or overchallenged when watching videos. Underchallenge tends to lead to passive viewing and is accompanied by the fact that the content conveyed is not elaborated more deeply and thus does not lead to further knowledge acquisition. This is particularly problematic with regard to illusions of knowledge, which can arise in particular with learning videos [91]. On the other hand, overchallenge due to a lack of prior knowledge when watching videos quickly leads to a loss of motivation to deal with the content. One way to reduce this strain is to use navigation options in the video, such as pausing or fast forward and rewinding, to promote individual knowledge acquisition [83]. 

Another way of supporting knowledge acquisition is the structured description of individual steps in complex problem-solving processes by experts (e.g., during a medical examination and diagnosis); when using worked examples, learners can focus better on the problem and the necessary solution steps than without this support (see also worked-examples effect [92,93]). The video used in the study described above showed the step-by-step approach of different professions in the interprofessional treatment of shoulder trauma [86]. The knowledge acquisition of the different professions while watching the video was also examined. It was shown that the different professions received content differently. Students of physiotherapy acquired knowledge relevant to medicine, which was presented in the video. For example, after watching the video they were able to understand the concrete anatomical course of various muscle strands in the shoulder better than before. Medical students, on the other hand, showed an increase in knowledge of physiotherapeutic topics, for example how to proceed in the physiotherapeutic follow-up treatment of shoulder trauma.

Taken together, the presentation of videos is also an option for addressing the four core competence domains of interprofessional collaboration. Interprofessional videos can be used for the development of values and ethics. Observational learning is particularly suitable for the acquisition of social behavior [85]. A video of a discussion on how an optimal treatment should be done could address different professional ethics and values. Students are more prone to adopt values when they follow a discussion where values are negotiated enthusiastically [94]. Instructional videos also offer the opportunity to raise awareness of profession-specific roles, competencies, and responsibilities. Videos can point out typical challenges of responsibilities and boundaries. Role problems are well known in interprofessional collaboration [14], and learners tend to overidentify with their own profession [95]. These issues can be explicitly addressed through role models that show appropriate interprofessional behavior, in particular when an optimal interprofessional collaboration is presented [96]. To improve the ability for interprofessional communication, videos can be used to present an ideal conversation among interprofessional role models. Common misunderstandings can be addressed directly by the role models in a video, which may support the establishment of a shared interprofessional language. Finally, videos may strengthen learners’ ability for interprofessional teamwork. For this purpose, videos need to be well scripted and include all professions that are relevant for a given medical case. 

## 4. Conclusions 

As explained above, social platforms and the use of videos offer great potential for interprofessional teaching in terms of supporting the four core competence dimensions. However, these approaches also have weaknesses that can be mutually compensated for. Accordingly, it is obvious to combine interprofessional videos with online-based interaction possibilities. This gives learners the opportunity to actively participate and exchange information with each other. A prerequisite for the active involvement of learners in digital interprofessional education are synchronous learning platforms where videos are streamed live at a specified time [97]. 

Through the use of audience response systems, learners can be given the opportunity to respond immediately to the questions asked in a live streamed video, which is perceived by learners as entertaining [98] and educational [99]. This may have a positive impact on the learning experience [98,100,101]. If the questions in the video are asked by representatives of different professions, active engagement with the questions of other professions can increase awareness of one’s own professional role, competences, and abilities. It can also improve awareness of one’s own limitations and the competence areas of the other professions [102,103]. A further advantage of a live broadcast is the possibility of a synchronous exchange among the learners about the content they have seen, for which chat tools can be used. According to contact theory [104], direct informal contact among learners offers the potential to learn from each other and to reduce existing negative stereotypes about other professions, resulting in a more positive image and appreciation of other professions [105,106]. This is particularly relevant for training in health-related professions, as the field is certainly prone to tribalism and traditional professional stereotypes that prevent an optimal treatment [14,15]. Different professions involved in patient care sometimes pursue different therapeutic approaches [107]. An appreciation of other professions can contribute to greater interprofessional tolerance that help to establish shared values that ultimately lead to more diverse approaches to solutions [108].

Promising approaches already exist, such as the interactive live format of the Sectio chirurgica platform (www.sectio-chirurgica.de). This is an online service that can be used by various medical professions. Depending on the respective clinical case, the interprofessional collaboration among various professions relevant to the case is presented in addition to anatomical content. Thus, learners have the opportunity to observe professional role models that discuss their opinions and treatment options in order to provide a shared best treatment idea. Learners can also observe how professionals deal with the challenges of differing terminology. Via a chat function, learners can actively contribute questions and comments to the event and exchange information with each other [109,110,111]. Originally, the platform was designed primarily for medical students, but students and trainees from various health care professions also use this course offer. Research on this format with a group of medical students showed that they had a higher increase in clinical knowledge when they watched a video showing interprofessional collaboration compared to students who received the same content from a representative of only one profession [111]. This platform serves as an optional supplementary instructional offer for health care students. Although students’ curriculum is quite dense, this offer is widely used, and it has been shown to improve their course performance [110]. In another study on interprofessional disaster management, the use of videos showing interprofessional collaboration contributed to a better understanding of the roles of learners from different professions [112]. 

Synchronous video-based teaching formats are disadvantageous with regard to the large organizational, technical, and financial effort involved, especially in direct comparison to classical text-based teaching material (Table 1). In particular, the creation of surgical or anatomical video material is associated with high costs. Only when the material is used repeatedly does a cost advantage arise in comparison to classical teaching. In order to enable an adequate interprofessional exchange, it must be guaranteed that a sufficient number of representatives of different professions view the educational material at the same time and exchange information about it, which requires a high level of organizational effort in preparation. Since synchronous chat activities are often used to discuss informal content among learners, knowledge transfer can suffer, which is why chat moderation is considered useful [113]. Also, frequently offered navigation options, such as fast forward, rewind, and pause for videos, have to be reconsidered when using a video on a synchronous learning platform, as they can make it difficult for learners to synchronously exchange interprofessional information about the contents of the video. However, not offering these navigation options is a challenge for individual learning. Nevertheless, we consider such synchronous video-based teaching formats to be extremely useful, especially in terms of promoting interprofessional understanding and collaboration among learners.

## 5. Outlook

Further research and theory-based developments are needed to explore the potential and limitations of digital media for the acquisition of skills relevant for interprofessional collaboration in the health sector. To this end, didactic teaching scenarios should be developed that combine established digital media (such as videos) suitable for knowledge transfer with digital synchronous tools that enable exchange among learners (e.g., via chat) or the joint processing of a task (e.g., via wikis, quizzes). Adequate formats would be synchronous, case-based webinars in which experts from different professions interact with each other using video-supported worked examples and for which tasks are set that learners from different professions must actively deal with. An example of such an interprofessional task would be the creation of a care plan that integrates all professions involved in the case. Promising approaches are currently being developed in which chats are offered synchronously with live videos and moderated in an interprofessional way. However, it should be noted that the possibilities for digitalization presented here make only small contributions to meeting the demand to implement interprofessional training approaches more firmly in medical education. Accordingly, they should rather be understood as a supplement to be used in combination with real interprofessional education. It is important to design such open learning formats in such a way that they can support interprofessional learning and interprofessional collaboration not only during medical education but also in later professional life in a clinical context.

The extent to which the technologies described and assessed here are actually capable of addressing the time and cost problems mentioned above remains to be critically examined. It is quite clear that the solution cannot be to simply add these digital approaches to the existing curriculum. This would only increase the time pressure for health care students. Teachers and curriculum designers must therefore ask themselves first and foremost how these offers can be integrated into the course of study in such a way that the problem is not made worse. This includes the possibility of adding interprofessional aspects to existing content or replacing current content with interprofessional topics. In this review, we have explained in detail under which circumstances this can be particularly successful and which technologies are suitable for which didactic goals. In this way, practitioners are given a guide on how they can integrate interprofessional approaches into medical training in a cost-effective way without overburdening their students.

## Figures and Tables

**Table 1:** Comparison of synchronous and asynchronous text- and video-based teaching formats.

Assessment Criteria		Social Interaction	Individual Knowledge Acquisition	Entertainment	Vividness of the Material	Organizational Effort	Flexibility	Costs
**Text-based**	Asynchronous	−	+	~	−	−	+	−
	Synchronous	+	−	~	−	−	−	~
**Video-based**	Asynchronous	−	+	+	+	+	+	~
	Synchronous	+	−	+	+	+	−	+

+ high|~ medium|− low.
